# Quality of Work: Validation of a New Instrument in Three Languages

**DOI:** 10.3390/ijerph121214958

**Published:** 2015-11-26

**Authors:** Georges Steffgen, Diane Kohl, Gerhard Reese, Christian Happ, Philipp Sischka

**Affiliations:** 1 Institute for Health and Behavior, University of Luxembourg, Esch-sur-Alzette L-4365, Luxembourg; diane.kohl@uni.lu (D.K.); philipp.sischka@uni.lu (P.S.); 2 Department of Social Psychology, Friedrich Schiller University Jena, Jena 07743, Germany; Gerhard.reese@uni-jena.de; 3 Department of Business Psychology, University of Trier, Trier 54286, Germany; happ@uni-trier.de

**Keywords:** quality of work, new questionnaire, well-being

## Abstract

*Introduction and objective:* A new instrument to measure quality of work was developed in three languages (German, French and Luxembourgish) and validated in a study of employees working in Luxembourg. *Methods and results:* A representative sample (*n* = 1529) was taken and exploratory factor analysis revealed a six-factor solution for the 21-item instrument (satisfaction and respect, mobbing, mental strain at work, cooperation, communication and feedback, and appraisal). Reliability analysis showed satisfying reliability for all six factors and the total questionnaire. In order to examine the construct validity of the new instrument, regression analyses were conducted to test whether the instrument predicted work characteristics’ influence on three components of well-being—burnout, psychological stress and maladaptive coping behaviors. *Conclusion:* The present validation offers a trilingual inventory for measuring quality of work that may be used, for example, as an assessment tool or for testing the effectiveness of interventions.

## 1. Introduction

There have been many changes in the labor market in recent decades, such as the delocalization of production, the development of non-permanent and part-time work, the introduction of new technologies and an increased demand of flexible employees with varied skills [1,2,3]. In order to deal efficiently with these new labor market developments, the European Union (EU) has devised different directives (for example Europe 2020), which are meant to promote a healthy and qualified labor force, high quality jobs and advanced training to ensure that employees are able to respond to these new challenges [4]. These directives also point out the necessity of extending purely economic work quality indicators like employment rates by psychosocial indicators that focus on the work itself. Thus, it seems timely to address these changes in the labor market in order to remedy the absence of such indicators. Therefore, we aimed to develop a new instrument that reflects an altered work reality: The Measuring Quality of Work questionnaire (MQW). In the following, we will present this new trilingual instrument and analyze its psychometric properties.

### 1.1. The Concept of Quality of Work

Clearly defining quality of work remains a challenge and the terms job quality, quality of work, and employment quality are often used interchangeably [5]. Quality in this sense refers to an appeal “to promote rising standards” [6]. Thus, measures of job quality, quality of work or quality of employment should not simply provide researchers with an existing overview of the employment situation, but also allow for an evaluation of the conditions uncovered. While conceptualizations vary within the social sciences, psychologists tend to focus on non-economic work factors such as intrinsically meaningful or challenging work, and in particular on the “goodness” of work when considering job quality [7]. Thus, definitions of job quality are often based on determinants of high-quality jobs. For example, Barling, Kelloway and Iverson define: “High-quality work provides the employee with the means (through extensive training) and the opportunity (…) to do great work” [8]. Job quality thus often focuses on salary, job security and fringe benefits, as they allow employees to pay bills and accumulate savings, as well as autonomy and control, as they provide employees with the opportunity to fulfill their own needs at work (e.g., fulfillment) [9]. On a general level, job quality is often assessed by aggregating different job components [7]. This way, one can explore whether job quality increases or decreases over time or if certain countries or work sectors produce a higher quantity of good jobs [10]. However, the disadvantage of this approach is that this clustering of job components that constitute a “good” job is a simplification and cannot show how jobs might differ on a broad array of characteristics [7]. For example, two jobs might be classified as “good” jobs, but might be good due to completely different characteristics. Thus, the concept of job quality to date seems to be too narrow.

Employment quality, when conceptualized different from quality of work, is usually seen as an extension of the latter. The European Foundation for the Improvement of Living and Working Conditions outlines employment quality as incorporating quality of work as well as variables about working conditions and social protection indicators [11]. Similarly, Johri [12] defines employment quality as including wider labor market characteristics and the match between employee and employment characteristics. The Commission of the European Communities proposes ten dimensions of employment quality such as inclusion and access to labor market [6]. Thus, the concept of employment quality goes beyond the concept of quality of work and includes general labor market characteristics (e.g., inequality). This approach is problematic, however, since changes in the labor market do not always go hand in hand with changes in quality of work. For example, at the labor market level, the creation of new temporary or fixed-term part-time jobs, might lead to an increase of inclusion or access to work, while at the same time leading to a decrease in purely work related characteristics (e.g., job security) [9,13]. Therefore, it seems appropriate and necessary to investigate the quality of work and labor market characteristics as separate constructs.

Quality of work bridges the gap between job quality and employment quality. Quality of work constitutes the activity of work itself and the conditions under which it takes place [13]. The authors therefore define it as a multidimensional concept concerned with the characteristics of work itself, as well as employees’ evaluation of these characteristics. This definition of quality of work includes both objective work characteristics (e.g., working hours) and subjective characteristics (e.g., satisfaction). It is furthermore closely related to the Job Demands-Resources (JD-R) model [14]. The JD-R model postulates that work characteristics can be divided into two groups: Job demands and job resources [15]. Job demands are physical, psychological, social or organizational job components that require continued physical and/or psychological effort [16]. Job resources on the other hand, are physical, psychological, social or organizational job components that are useful in reducing job demands and the associated negative consequences, useful in stimulating personal growth, learning and development and are functional in achieving work goals [15]. Consequently, quality of work comprises all the job demands and job resources employees encounter at work.

### 1.2. Measuring Quality of Work

Previous research on work quality has resulted in a plethora of indicators. The number of instruments somewhat reflects the diversity of conceptualizations of quality of work. Munoz de Bustillo and colleagues reviewed 18 existing work quality indicators (see Table 1) [13]. A detailed description of all these indicators is beyond the scope of this article. However, it is important to know that these indicators are diverse and were designed for multiple purposes. They range from six (e.g., Good and Bad Job Index [17]) to over one hundred variables (e.g., European Working Conditions Survey [18]), either focusing purely on work quality dimensions (e.g., European Job Quality Index [19], DGB Good Work Index, [20]) or including employment quality dimensions. Furthermore, the instruments Laeken [5], Indicators of Job Quality [21], Indicators of Quality in the Labor Market [22,23] and the Good and Bad Job Index [17] include longitudinal measures of job or income mobility and thus enable a comparison of quality of work over different time periods. However, Munoz de Bustillo and colleagues [13] also note the limitations of these existing indicators such as the inclusion and simultaneous evaluation of labor market characteristics not directly related to work. Since work components and labor market components rarely change at the same pace, indicators including both components may oversee or misinterpret important information. Furthermore, certain work quality dimensions that have shown to be important in research (e.g., intensity, wages) are absent in most of the indicators reviewed. This is problematic, as recent labor market research has called specifically for a survey of work intensification, the investigation of its origins, and its implication for workers [9]. Thus, while other indicators are useful for their specific purposes, a short worker-oriented instrument that bridges the gap between employment quality and job quality indicators, yet including previously neglected work components, is needed.

**Table 1:** Review of 18 existent quality of work instruments.

Name	Key Concepts	Reference
Laeken indicators of job quality (Laeken)	Income, Inequality, Material deprivation, Education, Health, Work/labor market	[5]
The European Job Quality Index (EJQI)	Wages, Non-standard forms of employment, Working time and work-life balance, Working conditions and job security, Skills and Career development, Collective interest representation and voice	[19]
European Working Conditions Survey (EWCS)	Working in Europe, Working environment and work organization, Quality of work and employment	[18]
Good Jobs Index (GJI)	Wages, income, poverty, Labor market segregation, Labor standards	[24]
Decent Work Index-1 (DWI-1)	Employment opportunities, Work conditions, Social security, Collective bargaining	[25]
Decent Work Index-2 (DWI-2)	Labor market security, Employment security	[26]
Decent Work Index-3 (DWI-3)	Economic equity	[27]
Decent Work Index-4 (DWI-4)	Economic democracy	[28]
Quality of Employment Indicators (QEI)	Work-life balance, Health and well-being, Skills development, Employment security	[29]
Indicators of Job Quality (IJQ)	Salary, Benefits, Work conditions, Well-being	[21]
Subjective Quality of Working Life Index (SQWLI)	Working conditions, Satisfaction, Security, Self-realization	[30]
DGB Good Work Index (DGBI)	Resources, Workload and stress, Income and security	[20]
Austrian Work Climate Index (WCI)	Economic and social change, Work conditions	[31]
Indicators of Quality of the Labor Market (IQL)	Employment quality	[22,23]
Quality of Work in Flanders (QWF)	Stress, Well-being, Learning opportunities, Work-life balance	[32]
Tangian’s proposal (Tangian)	Working conditions, Job quality	[33,34]
Good and Bad Jobs Index (GBJI)	Job satisfaction, Individual characteristics, Employer and workplace characteristics	[17]
Index of the characteristics related to the quality of employment (ICQE)	Employment quality, Enhanced capability, Labor market	[35]

### 1.3. Dimensions of Quality of Work

Identifying important dimensions of quality of work is usually done by one of two ways. Either employees are asked to indicate what is important to them in a job, or reasons why an employee left a job is connected to job components. On the one hand, evaluating changes in the quality of jobs over time in OECD countries showed that high income, flexible working hours, good opportunities for advancement, job security, having an interesting job, being able to work independently, being able to help other people and being useful to society were amongst the work components rated as very important [10]. On the other hand, importance of work components, as assessed by subsequent quit behavior, showed that job security, pay, hours and work itself were amongst the most important [36]. Similar results have also been found in relation to the JD-R model, where work overload, emotional demands, physical demands and work–home interference are typically listed as the most important job demands, while social support, relationship with supervisor, autonomy and feedback are listed as the most important job resources [15,37,38]. Job security is definitely an important work characteristic [39]. However, its measurement is often restricted to items inquiring about fear of losing one’s job. Yet, it is important to note, that employees also often fear losing their status within a company as well as losing out on an opportunity for promotion [40]. Importance of work and satisfaction with work are considered important intrinsic work rewards [10]. Work that is intrinsically important might lead to a reappraisal of work demands, thereby alleviating perceived work strain. Work intensity can influence employees negatively by causing them to fall behind on their workload or leading to mistakes, which then again can lead to negative supervisor-employee interactions [15].

The fact that some work components seem more important than others is also reflected in research on the relationship between work components and health. For instance, work intensification is usually associated with poor general health and poor quality of family relationships, while managerial support is associated with good health [9,40]. Poor social relationships at work, including mobbing, increase the risk of symptoms of depression, anxiety, and cardiovascular disease [41,42]. Some work components have a negative impact on their own, while others’ negative effect is mediated by other work characteristics. For example, jobs low in task discretion, where minimal training is needed to do a task, often lead to less job security as employees become replaceable [43]. Stressed employees might also unintentionally increase their work demands by working ineffectively and falling behind on their workload [15]. Similarly, social support and constructive feedback between colleagues or between supervisors and employees can provide emotional support, help finish a task in time and work more efficiently, thus easing the strain of work overload [15].

Overall, previous research suggests that there are recent changes in the labor market that need to be accounted for in contemporary quality of work research. Furthermore, the concept of quality of work needs additional clarification. Moreover, previous findings show that certain quality of work components impact health and well-being directly as well as indirectly via their influence on other work characteristics. The goal of our study thus was to develop a new quality of work instrument, which focuses on quality of work components that further influence other work characteristics, thereby making the instrument more concise. In the following, the development of this MQW questionnaire and the examination of its psychometric properties are presented (for the items see appendix Table A1).

## 2. Method

In a first step, we screened the scientific literature for relevant work characteristics. During this search, four dimensions were of importance: (a) the work characteristics should be in line with the definition of quality of work; (b) the work characteristics should be in line with the JD-R model; (c) the work characteristics should influence well-being and/or health; and (d) the work characteristics should influence other work outcomes. By focusing on these dimensions, we kept the instrument concise by examining those characteristics with the biggest impact on well-being and/or health. It was also important to include dimensions that have been largely absent from other instruments (e.g., work intensification) or dimensions that have not been measured entirely in the past (e.g., job security). Thus, in collaboration with experts from the Luxembourg Chamber of Labor (a council, which aims to defend the employees’ rights with regards to legislation) we developed 21 items, which cover the most important work characteristics, which influence other work components and affect well-being and/or health.

The 21 items were first developed in Luxembourgish by two people who were trilingual in the three languages (Luxembourgish, French and German) the questionnaire was supposed to be in. The French and the German versions were then created from the original Luxembourgish one by the same people. Using two trilingual people who are also aware of the cultural context of all three languages is based on several recommendations which aim at avoiding biases a single translator might have introduced [44,45]. After the initial translation the questionnaire was tested for comprehension and semantic meaning using five native speakers in each language. After the necessary adaptations were made, the items were tested in a study, which entailed computer assisted telephone interviews with 1529 employees working in Luxembourg. This study was intended to investigate quality of work as well as health and well-being in Luxembourg. Following data collection and preliminary data analysis, an exploratory factor analysis was performed. Finally regression analyses were used to investigate the criterion validity and the prediction power of the measure on employees’ well-being.

The representative sample contained 1529 employees working in Luxembourg. Sampling was based on gender, commuter status and work sector [46]. Included were Luxembourg residents and commuters from Belgium, France and Germany that received a financial payment regardless of hours per week worked. For ease of data collection, certain sectors were merged during sampling and analysis (e.g., industry, production, energy, and water were merged into the sector “industry and production”). The subsample answering the Luxembourgish version of the questionnaire made up 46.3% (Nlux = 708) of the whole sample, those answering the French version 41.6% (Nfr = 636) and those answering the German version 12.1% (Nge = 185). Throughout this article the analyses will be based on these three subsamples.

Table 2 shows the sample characteristics for this study. The mean age of the employees in the total sample was 45.71 years (SD = 8.37 years). Furthermore, 58.5% (*n* = 894) of the total sample was male. The majority of employees in the total sample either had a high school diploma (20.9%, *n* = 320) or an apprenticeship (33.3%, *n* = 509) and worked in either the public sector (12.2%, *n* = 186) or the finance sector (11.8%, *n* = 180). While the subsamples have similar characteristics to the total sample, there are some differences. As was to be expected, there are differences in the nationalities of the employees that have chosen to answer the different language versions of the questionnaire. Furthermore, slightly less people that have answered the French version have achieved a high school diploma or an apprenticeship as compared to the other two versions. Lastly, more people having answered the Luxembourgish version work in the public sector and less of them in the finance sector, while the opposite is true for the other two versions of the questionnaire.

**Table 2:** Sample characteristics.

Characteristic	Total Sample	Subsamples			Subsample Differences
		Luxembourgish	French	German	
*n*	1529	708	636	185	
Age (M; SD)	45.71; 8.37	46.10; 8.76 **^a^**	45.15; 7.97 **^a^**	46.13; 8.13 **^a^**	F (2, 1526) = 2.43
Gender					
Male (%)	58.5	58.5 **^a^**	58.0 **^a^**	60.0 **^a^**	χ^2^ (2) = 0.23
Female (%)	41.5	41.5 **^a^**	42.0 **^a^**	40.0 **^a^**	
Nationality					
Luxembourgish (%)	46.2	93.6	5.0	5.9	F (2, 1526) = 318.67 **
French (%)	22.4	0.4	53.5	0.0	
German (%)	10.5	1.1	0.2	82.2	
Educational Level					
High school diploma (%)	20.9	22.6 **^a^**	18.9	21.6 **^a^**	χ^2^ (2) = 21.36 **
Apprenticeship (%)	33.3	35.7 **^a^**	28.6	40.0 **^a^**	
Work sector					
Public sector (%)	12.2	22.3	3.3 **^a^**	3.8 **^a^**	χ^2^ (2) = 95.42 **
Finance (%)	11.8	8.9	14.2 **^a^**	14.6 **^a^**	

The **^a^** superscript denotes a subset of variables whose means and frequencies do not differ significantly from each other at 0.05 level (Bonferroni correction) ** *p* < 0.01

## 3. Results

In a first step, data were analyzed using exploratory factor analysis (EFA) to determine the latent factorial structure of the items and to identify factors with low loadings, which then may be dropped. Principal components analysis with varimax orthogonal rotation was used as it is independent of distributional assumptions, less likely to produce improper solutions and produces factors that are uncorrelated [47]. To ease comprehension and interpretation of the resulting factors, items have been re-examined and recoded if necessary. Table 3 shows the resulting factorial structure for the 21 items for the total sample.

EFAs of the subsamples confirmed the six-factor solution for all three subsamples. These six factors had a cumulative variance of 60.35% for the Luxembourgish subsample of the MQW, a cumulative variance of 60.12% for the French subsample and a cumulative variance of 64.73% for the German subsample. Overall reliability of the MQW is satisfying (α = 0.84) and this reliability is maintained for the three subsamples (Luxembourgish: α = 0.83, French: α = 0.85, German: α = 0.85).

### 3.1. Measures

Table 3 shows that the items load on six different factors. These six quality of work factors, will be described in more detail in the following. Reliability results are listed in Table 4.

**Table 3:** Factorial structure of the 21 items (total sample).

Items	Factors						Communalities
	1	2	3	4	5	6	
Satisfaction work	0.60						0.63
Satisfaction work conditions	0.74						0.62
Respects employees’ rights	0.72						0.62
Satisfaction work climate	0.73						0.64
Chose same work	0.65						0.49
Respect superior	0.51						0.54
Criticized by colleagues/superior		0.71					0.54
Ridiculed by colleagues/superior		0.73					0.57
Ignored by colleagues/superior		0.65					0.54
Assigned absurd duties		0.63					0.52
Conflicts at work		0.46					0.51
Concentrate on several activities			0.79				0.66
Intellectually demanding work			0.79				0.63
Work under pressure			0.63				0.58
Participation				0.74			0.59
Being informed at work				0.67			0.57
Feedback				0.59			0.45
Cooperation with colleagues					0.87		0.78
Support from colleagues					0.77		0.73
Work considered important						0.78	0.70
Work is appreciated						0.55	0.55
% variance explained	16.56	11.89	8.27	8.19	7.65	6.71	
Cumulative variance	16.56	28.45	36.71	44.90	52.55	59.26	

**Table 4:** Cronbach’s α for all the quality of work and well-being factors.

Factor	Total Sample (*n* = 1529)	Luxembourgish Sample (*n* = 708)	French Sample (*n* = 636)	German Sample (*n* = 185)
Satisfaction and respect	0.85	0.83	0.86	0.86
Mobbing	0.71	0.70	0.72	0.73
Mental strain at work	0.65	0.63	0.65	0.64
Communication and feedback	0.56	0.60	0.54	0.52
Cooperation	0.65	0.74	0.53	0.78
Appraisal	0.54	0.60	0.45	0.55
Burnout	0.79	0.53	0.55	0.58
Psychological stress	0.75	0.75	0.74	0.78
Coping	0.76	0.71	0.81	0.58

#### 3.1.1. Work Quality

*Satisfaction and respect* (6 items): The first factor evaluates an employee’s satisfaction with important work characteristics, such as work climate and work conditions. This is done directly by asking employees if they are satisfied with the work components in question and indirectly by asking employees if they would work a second time in the same company. Furthermore, this factor also assesses whether an employee feels respected at work. Again, this is done directly by asking employees if they are respected by their superior at work and indirectly by asking them if the company they work at respects its employees’ rights. The latter item also doubles as an indirect measure of job security. As was discussed in the introduction, job security also relates to the maintenance of certain job characteristics, such as wage and opportunity for promotion [39]. The authors argue that a company that respects its employees’ rights, is less likely to implement pay cuts or demote an employee. In line with the JD-R model, asking if employees are satisfied with their work climate and are respected by their superior helps evaluate the impact of social support on employees’ well-being [15]. Good relationships between colleagues and between an employee and his/her superior are important factors, which can potentially facilitate work, through working together to ease workload, for example. In addition, satisfaction with work itself can be an important component for work motivation, which in turn might lead to a reappraisal of job demands as challenges [15]. A high mean of “satisfaction and respect” shows that the employee is satisfied with and respected at work. The five-point Likert scale ranged from 1 (“not at all”) to 5 (“absolutely”). A sample item is “Are you satisfied with your work climate?”.

*Mobbing* (5 items): The second factor is concerned with mobbing behaviors experienced by an employee. This factor is made up of four items adapted from the Leymann Inventory of Psychological Terror (LIPT) [48]. The authors chose an item relating to attacks on social relations, namely being ignored at work by colleagues or a superior, an item relating to attacks on someone’s reputation, in particular being ridiculed at work and, finally, two items pertaining to attacks on the quality of work and work perspectives, *i.e.* being criticized in front of others at work, and being assigned absurd tasks. These items were included, as social support through colleagues or a superior at work is a powerful job resource that enables more efficient communication, better management of high workload through emotional support and physical help with work tasks [15]. Hence, mobbing negatively affects all of these work components. Furthermore, mobbing has been linked to various health complaints, such as depression, anxiety, and cardiovascular disease [41,42]. A fifth item is concerned with conflicts between an employee and people (s)he comes into contact with while at work, such as clients, students or patients, for instance. While these incidences might not be considered as classical mobbing, difficult or aggressive employee-client/patient/student interactions can have outcomes similar to classical mobbing behaviors [49]. This dimension has been recoded, so that a high means implies a positive outcome. Thus, a high mean of “mobbing” implies that an employee experiences little or no mobbing behaviors at work. The five-point Likert scale ranged from 1 (“very often”) to 5 (“never”). A sample item is “How often does your superior assign you duties that seem absurd?”.

*Mental strain at work* (3 items): The third factor is concerned with mental strain experienced at work. This factor is linked to work intensification, which has traditionally included time pressure and work overload [39]. Our three items cover having to work on different divers tasks at once, working under pressure and doing intellectually demanding work. Mental strain at work is an important job demand, as it drains employees physical and psychological resources [50]. Furthermore, the resulting stress and fatigue of prolonged mental strain at work and its consequences can lead employees to unintentionally add to their work demands, by working ineffectively and falling behind on their workload, for instance [15]. Work intensification has also been connected to negative health outcomes such as burnout [16]. The items about having to concentrate on multiple divers tasks at once and working under pressure, also double as task discretion items. The rationale is that given the choice, most employees would not freely chose to work on different divers tasks at once or to work under pressure. Furthermore, some research suggests that high work intensity jobs are also jobs with low task discretion [51]. Items in this factor have been recoded, so that a high mean points to a positive outcome. Thus, a high mean of “mental strain at work” signifies that an employee faces little mental strain at work. The five-point Likert scale ranged from 1 (“very often”) to 5 (“never”). A sample item is “How often do you work under pressure?”.

*Communication and Feedback* (3 items): The fourth factor aggregates items that relate to the communication between a company and the employee. Thus, this factor is concerned with whether an employee gets to participate in decision-making at work, whether the company informs him of future plans that the company has and whether an employee receives feedback from his superior or his colleagues. According to the JD-R model, communication between an employee and his/her work place is an important job resource. Efficient communication can ease workload if, for example, a superior gives clear instructions to an employee [15]. Furthermore, the authors argue that if an employee has a say in decisions taken by the company concerning his/her work, the resulting improvements could largely influence the quality of work of that employee. Being involved in the decisions made by the company and being well informed about future plans is also considered an important intrinsic job reward [52]. Research has shown that opportunities for an engagement in decision-making at work result in an increased quality of work [52]. A high mean of “communication and feedback” implies that an employee has ample opportunities to be involved in the decision-making process at work and received feedback from his work concerning future company plans and his own work. The five-point Likert scale ranged from 1 (“not at all”) to 5 (“absolutely”). A sample item is “Do you participate in the decisions made by your company?”.

*Cooperation* (2 items): The fifth factor relates to cooperation and social support between colleagues at work. One question asks directly whether an employee is supported by his/her colleagues at work. The second question also contains an element of task discretion. It enquires whether an employee cooperates with his/her colleagues at work. The rationale is that a person might prefer not to, chose not to, or be unable to cooperate with his/her colleagues. Social support and cooperation between colleagues can provide emotional support, help finish a task in time, and work more effectively, thus easing the strain of various job demands, such as work overload and being underqualified for a task [15]. A high mean of “cooperation” implies that the employee cooperates with and gets social support from others at work. The five-point Likert scale ranged from 1 (“not at all”) to 5 (“absolutely”). A sample item is “Do your colleagues support you at work?”.

*Appraisal* (2 items): The last factor aggregates two items which are concerned with an employee’s appraisal of work. These two questions relate to intrinsic job rewards such as whether an employee considers his/her work to be important or appreciated by the company. Intrinsic job rewards have an important impact on motivation. An employee that considers his/her work to be important and or appreciated by the company might reconsider certain job demands such as workload as a challenge rather than an issue, for example [15]. As such, our appraisal dimension indirectly impacts most work components, such as workload, time pressure and long or irregular work hours, for instance. A high mean of “appraisal” implies that an employee feels that his/her work is important and appreciated by his/her company. The five-point Likert scale ranged from 1 (“not at all”) to 5 (“absolutely”). A sample item is “Is your work appreciated by your company?”.

Thus, the EFA showed a six factors solution. The predictive value for well-being, as defined by burnout, psychological stress and coping behaviors of the MQW questionnaire, will be tested next.

#### 3.1.2. Well-Being

In order to calculate the criterion validity of this new instrument, the authors also used three scales from the study in order to investigate if the quality of work dimensions can be used to predict an employee’s well-being. In line with previous research, in this article well-being is made up of the dimensions burnout, psychological stress and maladaptive coping behaviors. These three scales will be described in the following section.

*Burnout* (7 items): The burnout scale is based on the classical burnout description by Maslach, Jackson and Leiter [53]. Thus, the items enquire about experiences of exhaustion, cynicism and professional efficacy. Exhaustion is characterized as lack of energy and feelings of chronic fatigue or strain [16]. Cynicism refers to feelings of detachment or indifference towards work or feelings that one’s work is meaningless [38]. Finally, professional efficacy relates to reduced feelings of competency and achievement in relation to work [16]. This scale was recoded, so that a high mean implies a positive outcome. Thus, a high mean of “burnout” implies that an employee experiences very little burnout. The five-point Likert scale ranged from 1 (“very often”) to 5 (“never”). A sample item is “How often do you feel that you cannot master your job any longer?”.

*Psychological stress* (6 items): This factor refers to psychological consequences of job demands, such as feeling stressed by work, feelings of frustration and not being able to let go of work even after work hours. Psychological stress is included in our well-being concept as is seen as one possible outcome if there is an imbalance of job demands and job resources [54]. Thus, chronic job demands lead to a health impairment process that drains employees’ resources and energy leading to a deterioration of health [16]. This deterioration of health often results in psychological health issues [55]. This scale was recoded, so that a high mean implies a positive outcome. Thus, a high mean of “psychological stress” signifies that an employee faces little psychological stress related to work. The five-point Likert scale ranged from 1 (“absolutely”) to 5 (“not at all”). A sample item is “Are you feeling stressed because of your work?”.

*Coping* (4 items): The coping scale asks about the use of maladaptive coping behaviors to deal with work related stress. Thus, the questions refer to the use of alcohol and drugs. Coping was included in our well-being concept, since work characteristics can impact well-being indirectly via behavior-change [56]. This can happen for two reasons: An employee might feel the need to use enhancing drugs to be able to cope with long work hours or a high workload [2]. Conversely, an employee might feel the need to use substances, such as alcohol or prescribed medication to be able to relax after long work hours or intense work pressures [2]. This use of substances can then in turn negatively affect well-being. This scale was recoded, so that a high mean implies a positive outcome. Thus, a high mean of “coping” implies that an employee seldom uses adaptive coping strategies to deal with work related stress. The five-point Likert scale ranged from 1 (“very often”) to 5 (“never”). A sample item is “How often have you called in sick to avoid going to work?”.

### 3.2. Construct Validity

Table 5 shows the descriptive statistics of the six quality of work factors. These results are similar to the results at the item level, which are therefore not included in this article. Keeping in mind the recoding of the items, the means show that, in general, employees in Luxembourg describe a “good” quality of work. The low mean of the “mental strain at work” factor implies that there is considerable mental strain at work. The means for the Luxembourgish and German subsamples are somewhat below the mean of the total sample, while the mean of the French subsample is somewhat above the mean of the total sample. However, participants answering the French version indicated fewer opportunities for communication and feedback. An ANOVA showed that there are no significant differences between the means of the different subsamples F (3,12) = 0.00 (*p* < 0.05).

**Table 5:** Means, Standard Deviations and Correlations between the six factors.

Factors	Correlations						
Total Sample (*n* = 1529)	M	SD	(1)	(2)	(3)	(4)	(5)
1. Satisfaction and respect	4.09	0.74					
2. Mobbing	4.22	0.57	0.57 **				
3. Mental strain at work	2.16	0.85	0.17 **	0.23 **			
4. Communication and feedback	3.45	10.04	0.45 **	0.24 **	−0.16 **		
5. Cooperation	4.52	0.72	0.38 **	0.27 **	−0.00	0.25 **	
6. Appraisal	4.45	0.68	0.58 **	0.37 **	0.02	0.36 **	0.31 **
Luxembourgish sample (*n* = 708)							
1. Satisfaction and respect	4.08	0.71					
2. Mobbing	4.22	0.56	0.55 **				
3. Mental strain at work	2.10	0.82	0.11 **	0.21 **			
4. Communication and feedback	3.58	0.99	0.43 **	0.17 **	−0.16 **		
5. Cooperation	4.53	0.70	0.38 **	0.25 **	−0.06	0.27 **	
6. Appraisal	4.37	0.72	0.64 **	0.35 **	−0.02	0.44 **	0.37 **
French sample (*n* = 636)							
1. Satisfaction and Respect	4.09	0.79					
2. Mobbing	4.21	0.59	0.61 **				
3. Mental strain at work	2.23	0.88	0.21 **	0.26 **			
4. Communication and Feedback	3.29	10.11	0.48 **	0.30 **	−0.15 **		
5. Cooperation	4.51	0.77	0.39 **	0.33 **	0.03	0.25 **	
6. Appraisal	4.57	0.61	0.52 **	0.41 **	0.03	0.30 **	0.29 **
German sample (*n* = 185)							
1. Satisfaction and Respect	4.09	0.66					
2. Mobbing	4.25	0.50	0.54 **				
3. Mental strain at work	2.07	0.74	0.21 **	0.21 **			
4. Communication and Feedback	3.55	0.93	0.39 **	0.35 **	−0.05		
5. Cooperation	4.52	0.66	0.26 **	0.10	0.08	0.21 **	
6. Appraisal	4.34	0.70	0.66 **	0.45 **	0.04	0.45 **	0.21 **

** *p* < 0.01.

Most factors in the total sample and the subsamples correlate significantly and positively with each other. Only “cooperation” and “appraisal” do not correlate with “mental strain at work”. Thus, mental strain experienced at work seems independent of the level of cooperation at or appraisal of work. Furthermore, the table shows that “communication and feedback” correlate negatively with “mental strain at work”. Thus, as mental strain at work decreases (a high mean implying less mental strain) communication and feedback also decrease (a low mean implying less communication and feedback) and vice versa. The only difference is the German subsample. Here, “communication and feedback” does not correlate significantly with “mental strain at work”. Another difference in the German subsample is that “cooperation” does not correlate significantly with “mobbing”.

In order to examine the construct validity of the MQW, three hierarchical regression analyses were calculated using SPSS 20, one with burnout as the outcome variable, one with psychological stress as the outcome variable and one with coping as the outcome variable. In step 1, gender, age and work sector were entered in the model. In step 2, the quality of work factors were entered in the model. All variables have been z-standardized.

Table 6 summarizes the hierarchical regression results for the outcome variables “burnout”, “psychological stress” and “coping”. Concerning the outcome variable “burnout” it appears that the results from the Luxembourgish subsample are somewhat closer to that of the French subsample, even though all three subsamples have similar results. In all regression models, “satisfaction and respect”, “mobbing” and “mental strain” significantly predicted burnout in employees working in Luxembourg. The results of the regression indicate that the predictors explained 38% (*R*^2^ = 0.38, F (9) = 33.16, *p* < 0.01) of the variance in the Luxembourg subsample, 42% (*R*^2^ = 0.42, F (9) = 34.25, *p* < 0.01) in the French and 44% (*R*^2^ = 0.44, F (9) = 10.42, *p* < 0.01) in the German subsample.

**Table 6:** Results of the hierarchical regression analyses.

Outcome Variable	Predictor	Total Sample		Luxembourgish Sample		French Sample		German Sample	
		b	t	b	t	b	t	b	t
**burnout**	Step 1	*n* = 1450		*n* = 683		*n* = 592		*n* = 175	
	Constant	0.24	2.87 **	0.26	2.00 *	0.06	0.42	0.49	2.21 *
	Age	0.08	3.07 **	0.08	2.33 *	0.05	1.14	0.07	0.93
	Gender	−0.8	−3.27 **	−0.20	−2.51 **	−0.07	−0.84	−0.26	−1.62
	Work sector								
	Production (omitted)								
	Personal services	0.21	2.78 *	0.35	3.26 **	−0.06	−0.49	0.07	0.33
	Commercial services	−0.03	−0.40	0.19	1.98	−0.44	−4.00 **	−0.08	−0.42
	IT and natural science services	−0.11	−0.92	−0.18	−0.90	−0.24	−1.51	0.80	2.43 *
	Others	0.05	0.48	0.11	0.80	0.02	0.13	−0.11	−0.40
		*R*^2^ = 0.02; F = 4.87 **		*R*^2^ = 0.03 F = 3.88 **		*R*^2^ = 0.04; F = 3.73 **		*R*^2^ = 0.06; F = 1.80	
	Step 2	*n* = 1450		*n* = 675		*n* = 580		*n* = 172	
	Constant	0.33	4.78 **	0.38	3.43 **	0.08	1.25	0.71	3.90 **
	Age	0.03	1.56	0.02	0.55	0.04	1.25	−0.05	−0.74
	Gender	−0.25	−5.50 **	−0.27	−4.00 **	−0.12	−1.63	−0.42	−3.11 **
	Work sector								
	Production (omitted)								
	Personal services	0.22	3.60 **	0.28	3.22 **	0.03	0.24	0.16	0.89
	Commercial services	0.02	0.38	0.20	2.56 *	−0.30	−3.71 **	−0.02	−0.14
	IT and natural science services	−0.01	−0.12	−0.02	−0.15	−0.11	−0.86	0.42	1.59
	Others	−0.02	−0.31	0.04	0.35	−0.12	−1.03	0.12	0.48
	Satisfaction and respect	0.29	9.03 **	0.26	5.46 **	0.33	6.85 **	0.22	2.27 *
	Mobbing	0.32	12.06 **	0.31	8.46 **	0.29	7.11 **	0.42	5.08 **
	Mental strain	0.14	5.91 **	0.12	3.67 **	0.14	3.82 **	0.16	2.21 *
	Communication and feedback	0.04	1.59	0.05	1.21	−0.01	−0.28	−0.03	−0.13
	Cooperation	0.03	1.36	0.02	0.64	0.02	0.53	0.13	2.01 *
	Appraisal	0.00	0.10	0.05	1.29	0.01	0.23	0.05	0.64
		*R*^2^ = 0.38; F = 71.71 **		*R*^2^ = 0.38; F = 33.16 **		*R*^2^ = 0.42; F = 34.25 **		*R*^2^ = 0.44; F = 10.42 **	
**psychological stress**	Step 1	*n* = 1450		*n* = 683		*n* = 593		*n* = 175	
	Constant	0.02	0.17	0.1	0.69	−0.04	−0.31	−0.31	−1.30
	Age	0.07	2.73 **	0.07	2.01 *	0.09	2.02 *	0.00	0.03
	Gender	−0.02	−0.32	−0.14	−1.66 *	0.09	1.01	0.17	1.01
	Work sector								
	Production (omitted)								
	Personal services	0.01	0.11	0.13	1.13	−0.15	−1.19	0.20	0.89
	Commercial services	−0.02	−0.27	0.17	1.61	−0.36	−3.31 **	0.12	0.55
	IT and natural science services	−0.17	−1.41	−0.28	−1.34	−0.29	−1.85	0.61	1.75
	Others	0.19	2.04 *	0.25	1.64	0.14	0.99	0.22	0.73
		*R*^2^ = 0.01; F = 2.65 *		*R*^2^ = 0.02; F = 2.68 *		*R*^2^ = 0.04; F =3.63 **		*R*^2^ = 0.04; F = 1.02	
	Step 2	*n* = 1450		*n* = 675		*n* = 581		*n* = 172	
	Constant	0.13	1.78	0.29	2.54 *	−0.04	−0.39	−0.07	−0.37
	Age	0.04	1.71	0.02	0.78	0.09	2.34 *	−0.11	−1.73
	Gender	−0.10	−2.20 *	−0.23	−3.31 **	0.01	0.09	0.02	0.15
	Work sector								
	Production (omitted)								
	Personal services	0.04	0.69	0.07	0.83	−0.07	−0.65	0.25	1.29
	Commercial services	0.05	0.82	0.13	1.54	−0.23	−2.46 *	0.22	1.38
	IT and natural science services	−0.03	−0.32	−0.06	−0.34	−0.12	−0.93	0.25	0.90
	Others	0.02	0.27	0.06	0.50	−0.05	−0.47	0.42	1.60
	Satisfaction and respect	0.21	6.51 **	0.28	5.83 **	0.16	3.30 **	0.26	2.47 *
	Mobbing	0.30	11.12 **	0.31	8.24 **	0.28	6.53 **	0.34	3.94 **
	Mental strain	0.33	13.78 **	0.34	9.96 **	0.30	7.79 **	0.32	4.23 **
	Communication and feedback	−0.03	−1.34	−0.05	−1.29	−0.03	−0.65	−0.07	−0.81
	Cooperation	0.04	1.52	0.01	0.31	0.07	1.84	0.03	0.40
	Appraisal	−0.02	−0.63	0.01	0.13	−0.09	−1.90	0.08	0.93
		*R*^2^ = 0.36; F = 67.16 **		*R*^2^ = 0.41; F = 38.71 **		*R*^2^ = 0.35; F = 25.03 **		*R*^2^ = 0.41; F = 9.11 **	
**coping**	Step 1	*n* = 1450		*n* = 683		*n* = 592		*n* = 175	
	Constant	0.02	0.23	−0.02	−0.12	0.03	0.21	0.05	0.32
	Age	0.02	0.58	0.02	0.47	0.01	0.21	−0.01	−0.25
	Gender	0.01	0.18	0.05	0.69	−0.03	−0.26	0.05	0.37
	Work sector								
	Production (omitted)								
	Personal services	−0.00	−0.04	0.08	0.86	−0.18	−1.20	−0.19	−1.17
	Commercial services	−0.07	−1.04	−0.02	−0.23	−0.20	−1.53	0.01	0.07
	IT and natural science services	0.02	0.15	0.08	0.45	−0.03	−0.15	0.16	0.62
	Others	−0.11	−1.12	−0.12	−0.90	−0.07	−0.42	−0.18	−0.82
		*R*^2^ = 0.00; F = 0.46		*R*^2^ = 0.01; F = 0.83		*R*^2^ = 0.01; F = 0.59		*R*^2^ = 0.02; F = 0.50	
	Step 2	*n* = 1450		*n* = 675		*n* = 580		*n* = 172	
	Constant	0.09	1.02	0.03	0.24	0.09	0.56	0.15	0.89
	Age	−0.00	0.65	−0.00	−0.13	0.01	0.09	−0.07	−1.35
	Gender	−0.04	−0.65	0.02	0.29	−0.07	−0.65	−0.02	−0.15
	Work sector								
	Production (omitted)								
	Personal services	−0.01	−0.18	0.05	0.57	−0.18	−1.17	−0.15	−0.94
	Commercial services	−0.06	−0.93	−0.01	−0.15	−0.17	−1.30	0.06	0.46
	IT and natural science services	0.04	0.38	0.09	0.51	0.03	0.15	0.04	0.15
	Others	−0.14	−1.43	−0.12	−0.93	−0.13	−0.80	−0.17	−0.78
	Satisfaction and respect	0.03	0.84	−0.01	−0.28	0.07	0.97	−0.03	−0.30
	Mobbing	0.23	6.95 **	0.16	4.03 **	0.28	4.51 **	0.30	4.03 **
	Mental strain	−0.02	−0.69	−0.03	−0.70	−0.00	−0.03	−0.04	−0.63
	Communication and feedback	−0.03	−0.97	0.00	0.05	−0.09	−1.60	−0.12	−1.56
	Cooperation	0.04	1.48	0.05	1.22	0.05	1.06	−0.02	−0.34
	Appraisal	−0.02	−0.58	0.02	0.47	−0.07	−0.96	0.12	1.71
		*R*^2^ = 0.06; F = 7.40 **		*R*^2^ = 0.05; F = 2.80 **		*R*^2^ = 0.08; F = 3.81 **		*R*^2^ = 0.18; F = 2.84 **	

** *p* < 0.01; * *p* < 0.05.

Concerning psychological stress as the outcome variable, the results of the Luxembourg and German subsamples are more similar to one another than are the results of the French subsample. In all subsamples, “satisfaction and respect” and “mobbing” and “mental strain” significantly predict psychological stress. The results of the regression indicate that the predictors explained 41% (*R*^2^ = 0.41, F (9) = 38.71, *p* < 0.01) of the variance in the Luxembourg subsample, 35% (*R*^2^ = 0.35, F (9) = 25.03, *p* < 0.01) in the French and 41% (*R*^2^ = 0.41, F (9) = 9.11, *p* < 0.01) in the German subsample.

Concerning coping as the outcome variable, Table 6 shows that mobbing is the only significant predictor of coping in employees working in Luxembourg in all three subsamples. However, the variance explained in the German subsample is much higher than in the other two. The results of the regression indicate that this predictor explained 5% (*R*^2^ = 0.05, F (9) = 2.80, *p* < 0.01) of the variance in the Luxembourgish subsample, 8% (*R*^2^ = 0.08, F (9) = 3.81, *p* < 0.01) in the French and 18% (*R*^2^ = 0.18, F (9) = 2.84, *p* < 0.01) in the German subsample.

## 4. Discussion

A review of the quality of work literature and the current labor market changes has shown a need for a new concise quality of work instrument that uses a clear definition of quality of work, is rooted in the JD-R model, focuses on work characteristics that influence health and/or well-being and on work characteristics that tend to influence other work outcomes. In collaboration with experts from the Luxembourg Chamber of Labor, we developed such an instrument using 21 questions that focus on the most important work components, which tend to affect other work characteristics. Here, the first analyses of the MQW questionnaire are presented.

An EFA showed a six-factor solution for the 21 items of the MWQ questionnaire. This solution was confirmed in all three subsamples. The six factors reflect dimensions widely cited in the work quality and JD-R model literature: satisfaction and respect, mobbing, mental strain at work, communication and feedback, cooperation, and appraisal [9,10,15]. Reliability analyses on the different factors showed generally acceptable Cronbach’s alphas apart from “communication and feedback” (ranging from 0.52 to 0.60) and “appraisal” (ranging from 0.45 to 0.60). The overall reliability of the MQW questionnaire is satisfying (ranging from 0.83 to 0.85). Correlations at the item and at the factor level show mostly moderately high positive correlations. This could be seen as evidence for a mediation effect between different work characteristics. For example, research has shown that valuing employees’ rights (e.g., correctly promoting people) is effective in remedying mobbing behaviors [57]. Similarly, being respected by your superior and being satisfied with work both increase cooperation between colleagues, possibly through increased motivation and the creation of a positive social work environment. Only having intellectually demanding work did not correlate with many of the other items. The relative independence of this item might be due to the fact that the nature of a work activity might not be influenced much by other work characteristics.

Descriptive data analysis showed that employees in Luxembourg need to concentrate on multiple different activities at once and work under pressure. Furthermore, their work is intellectually demanding and they have limited possibilities to participate in the decision-making process at work. This is in line with other research, which shows that work intensification is still increasing [9]. Most of the factors are positively correlated apart from appraisal and cooperation, which appear to be independent of mental strain at work. This finding is unusual as it seems to contradict previous findings showing that cooperation can ease mental strain through co-workers sharing the workload and providing emotional support [15]. Correspondingly, appraisal was thought to ease mental strain through a positive re-appraisal of demands as challenges [15]. This finding can also be found at the item level, where the correlation showed that as mental strain at work decreases communication and feedback also decrease and vice versa. A possible explanation for this finding could be that activities that require a lot of communication tend to be more complex activities that therefore produce more mental strain [39]. This explanation is in line with our conceptualization of mental strain at work as working under pressure, having intellectual work and having to concentrate on multiple activities at once, or, perhaps, it is that communication itself that increase perceived mental strain. Some research has discovered that an increased need for cooperation between co-workers can increase the incidence of mobbing via the development of cliques [58]. We have not found evidence for this claim. The mobbing factor correlates positively with the cooperation factor. However, it is possible that the challenges of working closely with others and negotiating conflicts, work schedules and work activities leads to an increase in perceived mental strain. Lastly, if communication, feedback and cooperation takes the shape of frequent and long meetings that employees need to attend and prepare for, this could eventually lead to increased strain as they have to work under pressure and concentrate on multiple divers intellectually demanding activities at once to meet deadlines [40].

The separate regression analyses showed that, across the three subsamples, burnout is significantly predicted by “satisfaction and respect”, “mobbing” and “mental strain”. This is in line with previous research, which has identified work characteristics such as strain and workload, and organizational characteristics such as role ambiguity and social support as predictors of burnout [59,60]. Workload is meant to affect burnout mainly through the exhaustion component, as constant job demands keep depleting an employee’s resources, thereby leading to fatigue [61]. Mobbing may affect the other predictors as it tends to diminish productivity, thereby increasing mental strain, and inhibits cooperation and commitment, thereby decreasing general satisfaction at work [62]. Job satisfaction has previously been found to negatively correlate with the emotional exhaustion and depersonalization components of burnout [63]. Communication and feedback, and the respect component as conceptualized by us have not yet been thoroughly investigated in relation to burnout. However, recently research has linked values and fairness to burnout [64]. Values in this sense means the ideals that people have about work, while fairness implies a fair and respectful treatment of employees at work [61]. It was discovered that values influence burnout through distress that develops when job demands force employees to go against their values [61]. Fairness was mostly influencing burnout via rewards implying that the unfair distribution of monetary or other rewards eventually leads to burnout [64]. However, since the factors of communication and feedback, and the respect components of satisfaction and respect are conceptualized differently, it is unclear whether they influence burnout in the same way. Moreover, it is interesting to note that in the regression analysis appraisal of work does not have any predictive power in relation to burnout, since burnout is often associated with low organizational commitment [65].

Psychological stress is significantly predicted by “mobbing” and “mental strain” across all subsamples. Work strain is commonly associated with psychological stress. If an employee has to work under pressure or otherwise has a high workload, it can happen that (s)he finds it hard to detach from work even after office hours [66]. Thus, the strain from work is even experienced at home, thus quickly depleting employees’ mental and physical resources leading to low mood and fatigue, among other outcomes [50]. The link between mobbing experiences and psychological stress is well documented in the literature. Exposure to mobbing has been linked to an increased risk of depression, feelings of low self-confidence, increased levels of feeling nervous or strained and sleeping difficulties [67,68,69]. Furthermore, mobbing victims are more likely to report feeling anxious and experiencing a deterioration of their general mental health [41].

Finally, mobbing is the only significant predictor of coping behaviors in employees across all the subsamples. Experiencing mobbing makes an employee more likely to use maladaptive coping behaviors to deal with work stress. This finding is an interesting extension to previous results, which associate mobbing with adverse health effects, such as somatization and anxiety [41,70]. It appears that in order to deal with these health issues, employees resort to using prescription medicine, absenteeism, alcohol and drugs. Mobbing at work had previously been linked to the use of sleep-inducing drugs and sedatives [68]. It is interesting to note that mental strain is not identified as a predictor of maladaptive coping behaviors in employees. It had previously been discovered that enhancing or sedating drugs are often used to either manage intense job demands such as long hours or to unwind after exposure to job demands, respectively [2].

Overall, the measures we introduced seem to account for a substantial amount of variance in the well-being of employees in Luxembourg, as defined by burnout, psychological stress and coping behaviors and thus represents an important addition to the prevailing quality of work research.

### Limitations and Future Research

The analyses and results presented in this article have some limitations. First of all, two of the developed factors, namely “communication and feedback”, and “appraisal”, are not as reliable as desired. However, since the overall alpha of the instrument is satisfying, it appears that the six different factors measure quality of work properly. Moreover, a detailed statistical comparison of the results obtained from the different language versions of the questionnaire would allow a further validation of the distinct versions of the questionnaire. The mode of data collection in itself also has possible limitations. In this study, data were collected using computer assisted telephone interviews (CATI). CATIs have the advantage of being inexpensive, less time consuming and provide a wide geographical access [71]. However, a different mode of data collection might have lead to a more heterogeneous work sector distribution. In the future therefore mixing both CATI and other modes to collect data should be considered. Future research should also further investigate the finding that communication and feedback actually increase perceived mental strain. It would be particularly useful in this context to identify potential moderators and mediators. Furthermore, our results could be clarified if the different quality of work factors would be linked to specific components of burnout, coping and psychological stress, respectively. In addition, further research should also re-examine the predictors of maladaptive coping behaviors as the extended use of enhancing or sedating drugs, and alcohol often have adverse health effects. Finally, future research should use the MQW in cross-cultural studies to determine if the tool is replicable sound, or requires modification due to cultural differences and methodological artifacts.

## 5. Conclusions

All in all, the MQW questionnaire has shown great potential in quality of work research. So far it has been shown that it can identify important predictors of well-being in employees, that it can be used to map the current quality of work of employees and that it can identify mediating effects between different work characteristics.

## Appendix

**Table A1:** The 21 items and six factors of the MQW questionnaire.

1	Are you satisfied with your work?	Satisfaction and Respect	Quality of Work
2	Are you satisfied with your work conditions?		
3	Does your company or institution respect its employees’ rights?		
4	Are you satisfied with your work climate?		
5	If you had the choice, would you work a second time in the same company/institution?		
6	Are you respected by your superior at work?		
7	How often is your work criticized by your colleagues or your superior?	Mobbing	
8	How often are you ridiculed by your colleagues or your superior in front of others?		
9	How often are you ignored at work by your colleagues or your superior?		
10	How often does your superior assign you duties that seem absurd?		
11	How often do you have conflicts with colleagues or other people with whom you are in contact at work?		
12	How often do you have to concentrate yourself on several divers activities at once?	Mental strain at work	
13	How often is your work intellectually demanding (for instance: having to concentrate for a long time)?		
14	How often do you work under pressure?		
15	Do you participate in the decisions made by your company?	Communication and Feedback	
16	Are you informed of internal decisions, changes and development plans of your company?		
17	Do you receive feedback regarding your work from your superior or from your colleagues?		
18	Do you cooperate with your colleagues at work?	Cooperation	
19	Do you colleagues support you at work?		
20	Do you consider your work to be important?	Appraisal	
21	Is your work appreciated by your company?		
